# Traditional paddy field‐supported bird diversity ignored by forest‐focused protection of ecosystems in tropical China

**DOI:** 10.1002/ece3.11408

**Published:** 2024-05-16

**Authors:** Mingxia Zhang, Yuqing Xu, Jiabin Li, Jianbo Yang, Qiaoyan Wang, Qiaoli Lin, Qihai Zhou, Lin Wang

**Affiliations:** ^1^ Key Laboratory of Ecology of Rare and Endangered Species and Environmental Protection Guangxi Normal University, Ministry of Education Guilin China; ^2^ Guangxi Key Laboratory of Rare and Endangered Animal Ecology Guangxi Normal University Guilin China; ^3^ Southeast Asia Biodiversity Research Institute Chinese Academy of Sciences Mengla China; ^4^ Center for Integrative Conservation, Xishuangbanna Tropical Botanical Garden Chinese Academy of Sciences Mengla China; ^5^ College of Life Sciences Guangxi Normal University Guilin China; ^6^ Center for Mountain Futures, Kunming Institute of Botany Chinese Academy of Sciences Kunming Yunnan China; ^7^ University of Chinese Academy of Sciences Beijing China; ^8^ Xishuangbanna National Nature Reserve Jinghong China

**Keywords:** birds, forest land, functional guilds, nature reserve, paddy field, Xishuangbanna

## Abstract

Biodiversity in tropical regions is facing threats from agricultural expansion and intensification. Therefore, a promising future for local ecosystem conservation depends not only on traditional protected areas but also on well‐managed agricultural landscapes. In this study, we compared the ecological traits of bird species in paddy fields outside of protected areas and natural forests within the protected areas of Xishuangbanna, southern China. There were 148 species in total, of which 98 were in forests and 55 in paddy fields. The abundance of birds in paddy fields was 176 per kilometer, which was much higher than the 60 per kilometer in forests. There were 26 law‐protected species observed, half of which were found in each habitat. The main functional groups living in nature reserves are invertivores and frugivores, whereas paddy fields provide habitats for aquatic predator and granivore bird species. Our results indicate that paddy fields act as a refuge for wetland and grassland bird species when natural wetlands disappear, highlighting the urgent need to focus more on wetland protection and eco‐friendly agricultural schemes at the landscape scale in future conservation policies.

## Graphical abstract

Current protected areas harbor insectivores and frugivore bird species; traditional paddy field provide habitat for wetland/grassland species; landscape‐scale management framework is necessary for effective conservation.

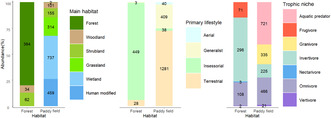

## 1 INTRODUCTION

The loss of biodiversity is a pressing global issue. Protected areas (PAs) around large primary forests are the cornerstone of conservation efforts. Although many PAs are located in remote areas to avoid conflict with human development (Venter et al., 2018), outside the PA system, ecosystem management to reconcile agricultural development and conservation is necessary at the landscape scale for sustainable development (Kremen & Merenlender, 2018; Scherr & McNeely, 2008). This is especially true in tropical areas, where natural resources are intensively used by humans (Sundar & Kittur, 2013).

Wetlands are important ecosystems that provide essential eco‐services such as food production, flood control, and maintenance of biodiversity. Paddy fields are human‐made wetlands that not only produce rice (*Oryza sativa*), a widely consumed staple food in Asia, but also have eco‐functions such as water purification and flood prevention (Natuhara, 2013). Paddy fields can also provide surrogate habitats for waterbirds when their natural wetlands disappear (Elphick, 2000). For some endangered species, such as the Australasian bittern (*Botaurus poiciloptilus*), paddy fields even host 40% of the global breeding population (Herring et al., 2019). The total area of paddy fields worldwide is approximately 130 million hectares (Yoon, 2009), with the largest production occurring in China. During the last 3 decades, the area of rice paddies has steadily decreased in southern China due to urbanization and infrastructure development and has increased in northeastern China (Xin et al., 2020).

In recent years, large areas of cash crops, such as rubber and oil palm, have expanded in tropical areas, replacing a large proportion of natural forest and other traditional agricultural land (Warren‐Thomas et al., 2018; Xu et al., 2022), For example, rubber plantations have been established on former natural forests or paddy fields along the China–Laos border and in northeastern Thailand (Liu et al., 2016; Mongkolsawat & Putklang, 2012). Many studies have shown that rubber and palm oil plantations support only limited eco‐functional groups compared with natural forests (Koh & Wilcove, 2008; Zhang et al., 2017). However, few studies have explored the biodiversity differences between traditional agricultural land and natural forests in tropical regions, although agricultural land could harbor different functional groups from natural forests and potentially act as “other effective area‐based conservation measures” in the entire conservation system (Hou et al., 2022). For example, when paddy fields are flooded at different frequencies after harvest, they can be used by different eco‐functional waterbird species (Strum et al., 2013).

Xishuangbanna, situated in southern China, is an important component of the Indo‐Burma biodiversity hotspot (Myers et al., 2000). Currently, the region is facing mounting conflicts between conservation and food security, making it imperative to integrate the livelihoods of local communities with conservation endeavors to safeguard indigenous biodiversity and foster regional sustainability (Zhang & Cao, 1995; Zhao et al., 2022). In the present study, we aimed to compare bird richness and abundance, including for threatened species, of forest and paddy field habitats, both within the protected area and in agricultural land of Xishuangbanna.

## 2 MATERIALS AND METHODS

### 2.1 Study area

The Xishuangbanna Dai Autonomous region is located between 21°10′–22°40′ N and 99°55′–101°50′ E, and the elevation ranges from 470 to 2430 m (Chen et al., 2016). The annual precipitation is 1317 mm, and >85% of the rainfall occurs during the wet season from May to October. Local farmers grew rice as a traditional livelihood; however, since the 1990s, most of them have switched to monoculture rubber plantations (Fu et al., 2010; Sturgeon & Menzies, 2006). Rubber plantations were first established in the 1950s in state farms, which were then expanded quickly to below 900 m asl after the 1990s among small local households, and even in some steep mountainous area higher than 900 m asl. The traditional paddy field is only maintained above 900 m asl, where rubber does not produce adequate lax, and hence is not profitable (Yi et al., 2014).

There are two National Nature Reserves (NNRs) in the region: the Nabanhe NNR (NNNR) and the Xishuangbanna National Nature Reserve (XNNR). The NNNR was built in 1991 around a watershed with an area of approximately 267 km^2^, and the XNNR was declared in 1959, with a total area of 2425 km^2^. XNNR comprises five separate sub‐regions: Mengla, Menglun, Mangao, Shangyong, and Mengyang. Both the NNRs were built to protect forest ecosystems and relevant rare species (Xishuangbanna Dai Autonomous Goverment, 2019). Although nature reserves are encroached upon by rubber plantations at low elevations, large areas of natural forests remain within the core areas (Chen et al., 2016). Limited grassland or open land was located inside the nature reserves. We identified 27 km^2^ (1% of total area) of wetland or grassland within the two NNRs based on remote sensing data. Most of them are smaller than 10 ha, with 18 km^2^ (0.6% of total area) of water. Most paddy fields in Xishuangbanna are maintained in Menghai County, where the average elevation is >900 m asl.

### 2.2 Field survey

**FIGURE 1:**
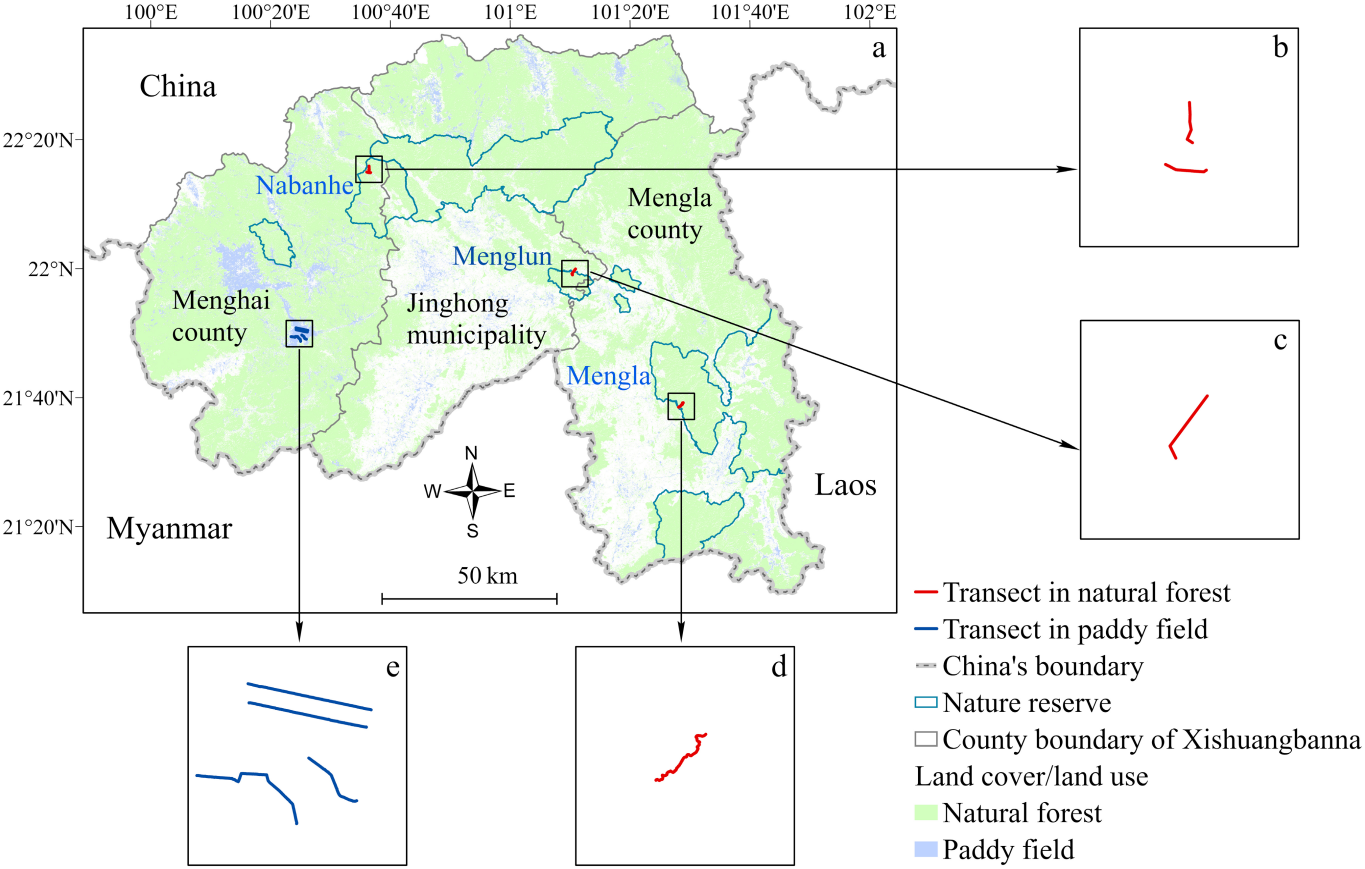
Selected bird survey transects in paddy fields and natural forests in Xishuangbanna, China.

We selected natural forests with similar elevation and topography to ensure their comparability with paddy fields. Four transects were allocated within the nature reserves as follows: two within NNNR, one within the Menglun sub‐region of XNNR, and one within the Mengla sub‐region of XNNR (Figure 1). The length of each transect was 1–3 km, and the elevation and terrain of all transects were checked in ArcGIS to ensure a similar condition between the paddy field and forest (Table S1). These transects lie at 1000–1100 m asl, and the terrain is plain. We interpreted the Landsat Operational Land Imager image to acquire land cover information. A random forest classifier that integrates phenology–topography and Landsat spectral data was used to achieve an overall accuracy of classification of 97.8% (Yang et al., 2021). The proportions of paddy fields and natural forests around an 800 m buffer area were estimated based on the interpreted image. The nearest distances away from a large forest patch (>100 ha) of every transect, were estimated to ensure similar landscape features among transects. Three of the authors performed the bird survey by walking along the transects at a speed of 1 km/h and recording all birds heard or seen during the survey (Gregory et al., 2004).

All transects were surveyed twice, once in the dry season and once in the wet season. The surveys were conducted during 8–11 AM or 3–7 PM (before sunset). Field surveys were conducted in June 2014, March 2017, and July 2018 to December 2019 for forest transects, and from April to October 2020 for paddy fields (Table S1). All the forest transects were located within a forest patch that is >100 ha.

### 2.3 Data analysis

The spatial autocorrelation among transects was assessed for all bird species using global Moran's I in ArcGIS. Rarefaction curves based on species richness were drawn to test whether the sampling effort was sufficient. The habitat type, trophic niche, and primary lifestyle of bird species were obtained from AVONET (Tobias et al., 2022). We then performed the χ^2^ test using R to compare the differences in bird feeding guilds between the forests and paddy fields.

## 3 RESULTS

No obvious spatial autocorrelation was detected among transects (Moran's I for abundance of forest = 0.70, z = 1.26, *p* = .21; Moran's I for abundance of paddy field = −0.87, z = −1.43, *p* = .15; Moran's I for richness of forest = 0.89, z = 1.37, *p* = .17; Moran's I for richness of paddy field = −0.42, z = −0.21, *p* = .84). The proportion of the paddy field area around paddy transects was 82–97% (89 ± 4), and the distance of paddy transects away from the large forest patch was 1179–1921 m (1,653 ± 171). The proportion of forests around forest transects was 75%–90% (84 ± 4).

**FIGURE 2:**
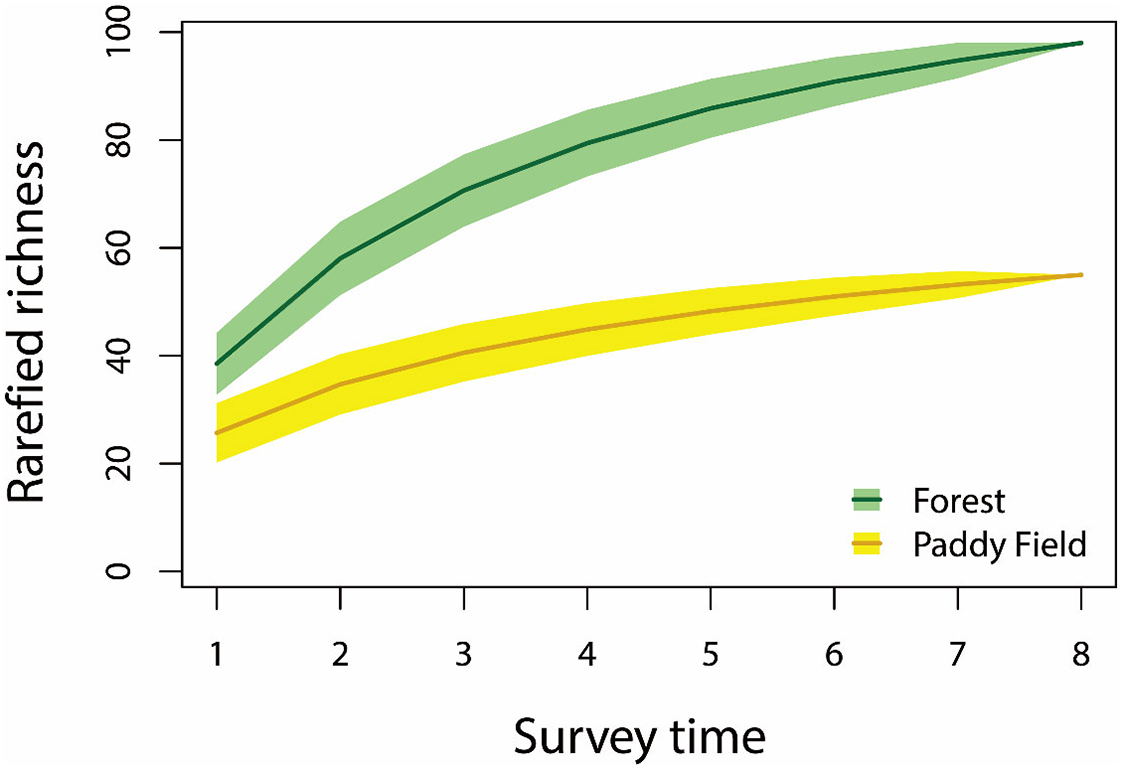
Accumulative curves of bird richness in forest and paddy field based on transect number in Xishuangbanna, China.

In total, 2248 birds of 148 species were recorded, of which 98 (480 individuals) were observed in forest transects, 55 (1768 individuals) in paddy fields, and 5 species were recorded in both habitat types (Table S2). The mean abundance per kilometer was 176 in paddy field and 60 in forest transects. We compared our survey results with the species recorded in *Bird Diversity in Xishuangbanna* (Luo et al., 2016), which compiled the bird records in this region from 1959 to 2013. Our result comprised 32% (145 of 469 species) of the historical record; three species were newly recorded: *Circus spilonotus*, *Garrulax strepitans*, and *Lewinia striata*. The rarefaction curve flattened for both forest and farmland (Figure 2).

One Class 1 (*Polyplectron bicalcaratum*) and 12 Class 2 key protected species, according to Chinese law, were recorded in forests (The NPC Standing Committee, 2016). Two Class 1 key protected species (*Emberiza aureola* and *Rostratula benghalensis*) and 11 Class 2 species were recorded in paddy fields. All the recorded birds were of the least concern status of IUCN, except for one critically endangered species, *Emberiza aureola*, in the paddy field (Table S2).

**FIGURE 3:**
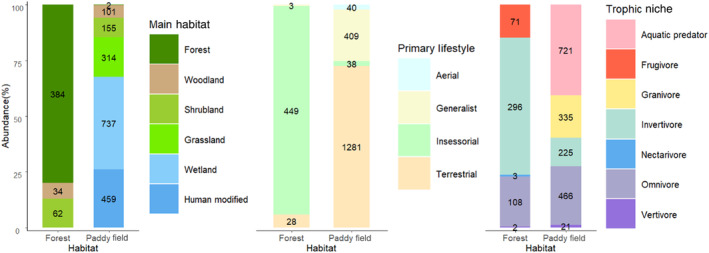
Abundance and percentage of bird species of different ecological traits in natural forests and paddy fields, Xishuangbanna, China.

The nectarivore and vertivore species were removed from the test of trophic niche as 21.4% of the species were <5. The test showed that habitat type (χ^2^ = 1820.965, *p* < .001), primary lifestyle (χ^2^ = 51.458, *p* < .001), and trophic niche (χ^2^ = 939.758, *p* < .001) were significantly different between the paddy fields and forests (Figure 3).

Granivores and aquatic predator species tended to live in paddy fields, whereas invertivores and frugivores preferred forests. The species that were found in the forest use the forest as the main habitat type, whereas 59% of species found in paddy fields use wetlands or grasslands as their main habitat type. Approximately 94% of species in forests are insessorial, and 73% of species in paddy fields are terrestrial. Finally, 77% of species in forests are invertivores and frugivores, while approximately 60% of species in paddy fields are aquatic predators or granivores.

## 4 DISCUSSION

Paddy fields sustain high diversity including for Odonata (Giuliano & Bogliani, 2019) and other arthropod species (Karenina et al., 2019), as well as many bird species (Elphick, 2000). Our study adds to the growing evidence for the conservation value of paddy fields. The results show that although the natural forest conserved in traditional nature reserves is critical for certain functional groups, such as insectivorous or frugivorous birds, agricultural land is important for wetland and grassland species, especially aquatic predator birds and granivores.

In north India, 99 species were observed in the agricultural wetland, and the species turnover is the main driver of local beta diversity, which indicates that all small wetland patches should be involved in conservation plans to reach the maximum protection efficiency (Sundar & Kittur, 2013). We calculated the landscape‐level variables, such as forest/paddy field area and the distance to a large forest patch. The results showed that all transects were located in large areas of corresponding type (forest or paddy field comprising >75% of the 800 m buffer area). However, we could not analyze the relationship between the bird diversity index and these variables as our sample sizes were small. In the future, more sampling efforts should be made to explore the relationships between different functional bird diversities and landscape‐level metrics.

We recorded an equal number (13) of law‐protected species within and outside the reserve, and observed that the abundance of species was much higher in paddy fields. This may be due to the higher detectability in the open environment. However, detectability bias was alleviated by recording both seen and heard species in the forest, which is a common way to record bird species in dense tropical forest (Haselmayer & Quinn, 2000; Thunhikorn et al., 2016). Several protected species in forests have loud and distinctive calls, such as *Aegithina lafresnayei*, *Polyplectron bicalcaratum*, *Psarisomus dalhousiae*, and *Harpactes erythrocephalus*. In the paddy field, we recorded most species only by vision. The higher abundance could be due to the higher density of raptors that prefer grassland (all raptors are protected by Chinese wildlife protection law), such as harrier (*Circus* spp.) and black‐shouldered kite (*Elanus caeruleus*), present in fallow land. We recorded two Class 1 species (*Emberiza aureola* and *Rostratula benghalensis*) in paddy fields, whereas only one Class 1 species (*Polyplectron bicalcaratum*) was recorded in the XNNR. Both *Emberiza aureola* and *Rostratula benghalensis* use paddy fields as their main habitat type. *Rostratula benghalensis* was shown to depend on fallow land in Japan and it underwent a drastic decline in Japan due to the loss of wet fallow fields after the 1990s (Katayama et al., 2020), indicating the importance of traditional ways of maintaining fallow fields being part of the ecological growth process.

Globally, natural wetlands have disappeared considerably under human development pressure (Giuliano & Bogliani, 2019; Sundar & Kittur, 2013). Compared with that for forest land, wetland conservation policy started later in China: the Forest Law of the People's Republic of China was firstly issued in 1984 (The NPC Standing Committee, 1984), while the Wetland Conservation Law of the People's Republic of China was issued in 2021 (The NPC Standing Committee, 2021). The wetland area kept decreasing during the past 4 decades, even within national wetland reserves (Meng et al., 2017; Zheng et al., 2012). Xishuangbanna once comprised a large area of wetland (Yan et al., 2022). The people of Xishuangbanna had a livelihood that was connected to the wetland. Wetland plants were used as medicine, food, and in religious ceremonies (Fang et al., 2006). Unfortunately, both NNRs in this region focus on tropical forest ecosystems and related wildlife (Xishuangbanna Dai Autonomous Goverment, 2019, 2023). Data on the rate of change in wetlands before 2008 were not available; however, the area of natural wetlands decreased considerably by 84.7% in Xishuangbanna during 2008–2017 (Yan et al., 2022) due to climate change, reclamation, and construction of reservoirs (Meng et al., 2017).

In tropical China, the disappearance of natural wetlands and the switch from paddy fields to rubber plantations have led to habitat loss for many wetland‐ and grassland‐dependent species. For example, the Sarus crane (*Grus antigone*) mainly uses natural wetlands or paddy fields as its habitat (Van Zalinge et al., 2023) and is only distributed in Yunnan Province in China. *Grus antigone* was observed in Xishuangbanna during 1959–1960, and later, it was reported by local people in 1987 (Yang, 1994). However, it has not been observed for >30 years after 1987; therefore, the species was recently designated as “regionally extinct” in China (Zhang et al., 2016).

Our results strongly suggest that more conservation efforts should be made toward wetland protection in the future. Xishuangbanna now is a protection site of migratory birds of China (National Forestry and Grassland Administration, 2022).An eco‐friendly agricultural scheme that combines eco‐tours and traditional culture, such as switching low‐profit rubber plantation above 900 m asl back to paddy fields, should be applied in Xishuangbanna and in other Southeast Asian regions that have similar environmental conditions. Given the emphasis on ecosystem protection and large‐scale conservation by the Chinese government in recent years, proper management at the landscape scale will hopefully integrate conservation and livelihoods of the local people. The ecological value of paddy fields could be maintained or augmented by applying several measures, such as management of flooding practice (Herring et al., 2019; Strum et al., 2013), building high‐diversity vegetation patches (Horgan et al., 2016), or enlarging ditchs (Giuliano & Bogliani, 2019) within the field. Eco‐friendly farming practices can also be boosted by developing wildlife‐friendly labels for the market (Mameno et al., 2021) or providing incentives for farmers to incorporate species conservation during rice growing (Herring et al., 2022).

## AUTHOR CONTRIBUTIONS

**Mingxia Zhang:** Conceptualization (lead); funding acquisition (equal); investigation (lead); writing – original draft (lead); writing – review and editing (equal). **Yuqing Xu:** Data curation (lead); funding acquisition (supporting); writing – original draft (equal). **Jiabin Li:** Investigation (supporting). **Jianbo Yang:** Data curation (supporting). **Qiaoyan Wang:** Investigation (supporting). **Qiaoli Lin:** Data curation (supporting). **Qihai Zhou:** Conceptualization (equal); funding acquisition (equal); writing – review and editing (lead). **Lin Wang:** Conceptualization (equal); funding acquisition (lead); writing – review and editing (equal).

## FUNDING INFORMATION

This study was supported by the Special Foundation for scholarship of Guangxi Science and Technology Program (Guike AD21220091), National Natural Science Foundation of China (grant numbers: 32260329, 32371745, and 32170492), the Biodiversity Investigation, Observation, and Assessment Program (2019–2023) of the Ministry of Ecology and Environment of China (grant number: 2019‐2‐4), Yunnan Province Science and Technology Department (grant number: 202203AP140007), 14th Five‐Year Plan of the Xishuangbanna Tropical Botanical Garden, Chinese Academy of Sciences (E3ZKFF7B, E3ZKFF9B), and Innovation Project of Guangxi Graduate Education (grant number: YCSW2022142).

## CONFLICT OF INTEREST STATEMENT

The authors declare no conflict of interest.

## Supporting information


Data S1.

## Data Availability

The data supporting the findings of this study are available in the supplementary material.
